# Coherent X-ray diffraction imaging of single particles: background impact on 3D reconstruction

**DOI:** 10.1107/S1600576724006101

**Published:** 2024-08-30

**Authors:** August Wollter, Tomas Ekeberg

**Affiliations:** a Department of Cell and Molecular Biology, Uppsala University, Husargatan 3, 75124Uppsala, Sweden; Uppsala University, Sweden; The European Extreme Light Infrastucture, Czechia

**Keywords:** X-ray free-electron lasers, structural biology, coherent diffractive imaging, background noise, EMC, phase retrieval

## Abstract

Coherent diffractive imaging with X-ray free-electron lasers could enable structural studies of macromolecules at room temperature. This type of experiment could provide a means to study structural dynamics on the femtosecond timescale. However, the diffraction from a single protein is weak compared with the incoherent scattering from background sources, which negatively affects the reconstruction analysis. This work evaluates the effects of the presence of background on the analysis pipeline. Background measurements from the European X-ray Free-Electron Laser were combined with simulated diffraction patterns and treated by a standard reconstruction procedure, including orientation recovery with the expand, maximize and compress algorithm and 3D phase retrieval. Background scattering did have an adverse effect on the estimated resolution of the reconstructed density maps. Still, the reconstructions generally worked when the signal-to-background ratio was 0.6 or better, in the momentum transfer shell of the highest reconstructed resolution. The results also suggest that the signal-to-background requirement increases at higher resolution. This study gives an indication of what is possible at current setups at X-ray free-electron lasers with regards to expected background strength and establishes a target for experimental optimization of the background.

## Abstract 2

Reconstruction methods for coherent diffractive imaging of single particles with ultrashort X-ray pulses were tested with various amounts of background noise, investigating the effect on resolution.

## 1. Introduction

### 1.1. Coherent diffractive imaging of single particles

With the extremely bright and short pulses produced by an X-ray free-electron laser (XFEL), it can be possible to measure diffraction from a single biological particle, such as a protein or virus. The particle will be severely photo-ionized, and the remaining positive charges will repel each other, which causes the particle to explode. The femtosecond pulses of the XFEL are, however, short enough that the X-rays will have left the sample before the time it takes for this damage to affect the structure. The measurement represents the undamaged particle—this is known as diffraction before destruction (Neutze *et al.*, 2000 ▸).

To inject the particles into the X-ray beam with precision, an electrospray injector (ESI) is commonly used (Bielecki *et al.*, 2019 ▸). An ESI works by applying a high voltage over the particle solution as it exits a capillary, and the excess charge will drive the formation of small droplets. Most of the buffer evaporates as the particle droplets travel toward the X-ray beam, leaving only a stream of dry particles. To protect the droplets from discharge, the capillary is surrounded by a constant flow of inert gases, like nitrogen and carbon dioxide. Some of this gas also enters the interaction region, and scattering from this gas is a major source of photon background and significantly harms the data analysis process. Scattering from the beamline elements, such as the apertures, is another source of background scattering.

Most proteins are small and weak scatterers, and even though the X-ray pulses are very bright the diffracted signal is still weak. The noisy background often overpowers the signal, especially at high scattering angles where high-resolution information is encoded. Structural reconstructions of small particles remain elusive, but there have been some successes with reconstructions of viruses and cells (Ekeberg *et al.*, 2015 ▸; Reddy *et al.*, 2017 ▸; van der Schot *et al.*, 2015 ▸; Ayyer *et al.*, 2021 ▸) and measurements of proteins (Ekeberg *et al.*, 2024 ▸).

In this paper, we test how the strength of this background and the strength of the signal itself affect the reconstruction pipeline, including orientation recovery and phase retrieval. The goal is that this should help us understand the requirements for future experiments.

### 1.2. The analysis pipeline

Coherent diffractive imaging is a lensless technique, and the real-space view of the particle is therefore not immediately available in the measured image, as it would be in, for example, cryogenic electron microscopy (cryo-EM). Rather, we measure the diffracted photons directly, similarly to X-ray crystallography. In X-ray crystallography, the repeated cells in the crystal structure amplify the signal in Bragg spots but cancel it elsewhere. This leads to a very high diffracted signal that is only sampled at a limited number of points. However, diffraction from a single particle would not have Bragg spots, so, provided that the signal is strong enough to be measurable without the amplification from the crystal, the diffraction signal can be measured at a much higher sampling density. This property can actually allow for the phase problem to be solved directly, using only constraints about the extent of the particle, as opposed to crystallography where redundant data or prior information about the structure is usually required (Sayre, 1991 ▸).

The phase problem is the task of performing an inverse Fourier transform without access to the complex phases and is found in many disparate fields like astronomy and X-ray crystallography. The task of reconstructing the electron density from only an intensity measurement falls into this category (Fienup, 1982 ▸). Because the object is limited in extent in real space and we sample the diffraction space at a sufficiently high sampling density, we can use iterative projection algorithms to recover the phases and overcome the phase problem. These are iterative algorithms that alternately apply constraints in real and Fourier space, thereby approaching phases that approximately satisfy both constraints. The simplest algorithm of this class is error reduction (ER) (Fienup, 1978 ▸), while other more involved approaches include hybrid input–output (Fienup, 1982 ▸) and relaxed averaged alternating reflections (RAAR) (Luke, 2005 ▸).

To recover a 3D electron density we need a series of diffraction measurements of the particle from different orientations. Each of these measurements contains information on the Ewald sphere surface that cuts through the Fourier transform of the particle, and many measurements, with the particle in different orientations, are needed to build the full 3D Fourier transform. If the particle orientations were known for each measurement, one could assemble a 3D intensity map directly, but in single-particle experiments, the particles are in random and unknown orientations. We therefore use an orientation recovery algorithm called expand, maximize and compress (EMC) to recover the orientations from the diffraction patterns (Loh & Elser, 2009 ▸).

EMC is based on expectation maximization and iteratively builds a 3D diffraction intensity model to infer relative orientations of the diffraction patterns. Initially, an intensity model is assembled from the diffraction patterns at random orientations, and then the diffraction patterns are exhaustively compared with the model at orientations taken from a uniform sampling of the 3D rotation group. This is done by first expanding the model into slices corresponding to the respective Ewald sphere for each considered orientation. One then calculates the conditional probability for each diffraction pattern to be measured given every specific slice in the expanded model. The next iterate is created by compressing the patterns into a new 3D intensity map, using the conditional probability as a weight for each orientation. This process is repeated for several iterations, either producing a model of increasing quality or sometimes collapsing into a model identifiable as a distinct fail-state such as all patterns being distributed equally across all orientations.

The output of EMC is the 3D diffraction intensity map. We can perform an iterative phase retrieval algorithm on this intensity map to recover a model of the electron density of the particle. In this paper, we investigate how the background strength impacts the resolution of the final electron-density map.

## 2. Methods

### 2.1. Experimental background and simulated diffraction

To test the impact of background noise on the orientation recovery and phase retrieval, we simulated diffraction images at different signal strengths. These were combined with measurements of the background obtained with the AGIPD detector (Henrich *et al.*, 2011 ▸) at the Single Particles, Clusters and Biomolecules (SPB) beamline at the European XFEL. The data were collected as part of a community experiment in October 2019.

An ESI was used without particles in the solution. Therefore, the sources of the scattering were solely the evaporated buffer, the carrier gas, a mix of nitrogen and carbon dioxide, and beamline elements. The detector data were corrected for gain and offset with the standard processing pipeline provided by the European XFEL, and the measurements were weak enough for the AGIPD to be in the high-gain mode for all pixels and patterns. We fitted a function consisting of two bell curves to the zero and first photon peaks of the histogram of the detector output. We then used the centres of the two bell curves as the scale to convert the detector units to photon counts

In this study, we wanted to cover a range of background strengths significantly weaker than the measured background, which was, on average, too high for our reconstruction. To downscale the backgrounds while preserving Poisson statistics, we randomly kept or discarded each photon in the background data set according to the desired intensity reduction. In this manner, we created 20 sets of background patterns of different strengths, ranging from 37 to 746 average photons per pattern in uniform steps, cropped to a 1024^2^ pixel array.

We simulated 9900 diffraction patterns of bacterial phytochrome, PDB entry 4o01 (Takala *et al.*, 2014 ▸), using the *Condor* software package (Hantke *et al.*, 2016 ▸). We used a photon energy of 8 keV and generated four data sets with different average signal strengths, ranging from 457 to 1826 photons per diffraction pattern. The detector had 1024^2^ pixels, with a pixel size of 110 µm, and a distance of 13 cm from the interaction region, which gives a full-period resolution of 3.84 Å at the edge of the detector. These patterns were masked to match the geometry of the AGIPD detector, which has gaps between panels.

We thus created 84 synthetic data sets, combining the four different signal strengths with the 20 different background strengths and an additional case without background.

### 2.2. Orientation recovery

We ran 45 iterations of EMC on our data sets after downsampling to 128^2^ pixels to strike a good balance between computational time and oversampling. The orientation recovery was repeated ten times for each data set, each time with a unique random starting map, to check for potential effects on the reproducibility of the algorithm. The orientation sampling included 50 100 possible orientations taken from the 3D rotation group as described by Loh & Elser (2009 ▸).

We also used a deterministic annealing scheme applied to the conditional probabilities, similar to the approach described by Ayyer *et al.* (2016 ▸) and Wollter *et al.* (2024 ▸). The conditional probabilities *R*_*d*_ for diffraction pattern *d* were exponentiated by β, 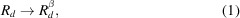
and the value of β was increased from 0.065 in the first iteration by 1.2% per iteration to 0.01 at iteration 38 and then remained constant. This was done to keep patterns from getting stuck in local maxima and to encourage EMC to converge more smoothly.

To evaluate each EMC run we compared the recovered orientations with the ground truth orientations in our simulations. Although the orientations that EMC recovers are internally consistent in a successful reconstruction, the recovered orientations can typically differ from the ground truth orientations by an unknown overall rotation, precluding a direct comparison. Instead, we considered the average relative orientation error, previously described by Wollter *et al.* (2024 ▸), but in this case including particle symmetry as well.

The average relative orientation error is an error metric for EMC that takes advantage of the fact that relative orientations are preserved over the global rotation between the recovered and ground truth orientations. For a pair of patterns with recovered orientations *a* and *b*, their relative rotation *r* = *ba*^−1^ would be identical to the relative rotation of the corresponding ground truth orientations, *r*′ = *b*′*a*′^−1^, if the orientation recovery was perfect. We therefore used the difference between *r* and *r*′ as a quality measurement for the orientation recovery, 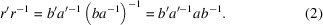
The twofold rotational symmetry of bacterial phytochrome had to be taken into account because EMC could recover the symmetric partner of any rotations. The metric therefore includes checking all permutations to find the one that minimizes the error. We calculated the average of the angle of the relative orientation error for *k* randomly selected pairs of orientations:
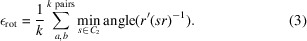
We get the average relative orientation error, ε_rot_, where 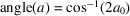, *a*_0_ is the first element of a quaternion describing rotation *a* and *s* is an element of the symmetry group of the particle, which for bacterial phytochrome is *C*_2_. We considered *k* = 1000 pairs of orientations.

The recovered orientations that achieved the lowest ε_rot_ in EMC, out of the ten independent reconstructions, were selected for phase retrieval. This was done to give all phase retrievals a good starting point, so comparisons between the reconstructions would be more meaningful. The results thus represent an ideal outcome from a data set, rather than an average.

### 2.3. Phase retrieval

For phase retrieval, we used the recovered orientations from EMC and assembled patterns which were only downsampled to 256^2^ pixels into more detailed 3D intensity maps compared with the direct output from EMC. We found that, when keeping the original sampling of 128^2^ pixels from EMC, the results of the phase retrieval never converged to reasonable orientations. Since phase retrieval is significantly cheaper computationally, the larger patterns did not pose a problem for this analysis.

Because the background noise was incoherently added to the signal patterns, the background photons contributed a spherically symmetric function to the 3D intensity. All attempts to run the phase retrieval algorithm without taking this background noise into account failed. Therefore we subtracted the radial average of the background from the 3D intensity before phase retrieval, which did allow for successful phase retrieval. In our case, this background distribution was known exactly, but during an experiment it could be measured separately, either by running the injector without sample or by averaging diffraction patterns classified as containing no sample. Ayyer *et al.* (2019 ▸) demonstrated how the background function can be estimated iteratively during phase retrieval, so even if the background distribution is not measured it can be possible to deduce and subtract it after the experiment.

For each combination of background and signal strength, we performed 100 independent phase retrievals. We started with 10 000 iterations of RAAR using the shrinkwrap algorithm to optimize the real-space constraint (Marchesini *et al.*, 2003 ▸). Every 100 iterations we updated the real-space support area, using a linear interpolation starting from 1% of the total area to 0.2% at the final iteration. The standard deviation of the Gaussian blur was similarly interpolated from 2 to 1 pixels. After RAAR, we refined with 2500 iterations of ER, using the final support from shrinkwrap as a static real-space support.

To evaluate the phase retrieval we considered the phase retrieval transfer function (PRTF) (Chapman *et al.*, 2006 ▸). The PRTF is a measure of the reproducibility of the recovered phases. If the 100 independent phase retrievals are in agreement for a given pixel then the value of the PRTF in that pixel will be one, and if the phases are uncorrelated the value will be closer to zero. To estimate the resolution of a reconstruction the radial average of the PRTF is calculated, and the momentum transfer at which this radial average falls below the threshold *e*^−1^ is identified. The resolution is then said to be the inverse of this momentum transfer.

We validate the resolution from the PRTF by calculating the Fourier shell correlation (FSC) between the Fourier transform of the average density map from the 100 reconstructions and the Fourier transform of the ground truth density. The FSC is a normalized correlation factor between two complex maps and is normally used to compare reconstructions from two different subsets of a data set. Instead, we compare the average map with the ground truth density, after aligning the average map using *ChimeraX*’s fit-in-map feature (Goddard *et al.*, 2018 ▸), to measure how close the reconstructions were to the actual density.

The resolution estimate from an FSC is usually defined as where it falls below the ½-bit threshold. The ½-bit threshold depends on the number of voxels in the considered shell and is derived by assuming that both data subsets have a noise and a signal contribution (van Heel & Schatz, 2005 ▸). In our case, we have no noise contribution in the ground truth and the ½-bit threshold is therefore slightly stricter than for the half-data-set comparison. Because of the differences, the resolution we report here is not directly comparable with the resolution from a PRTF or the FSC from a cryo-EM experiment, but they would be expected to be in a similar range and follow similar trends.

## 3. Results

**Figure 1:**
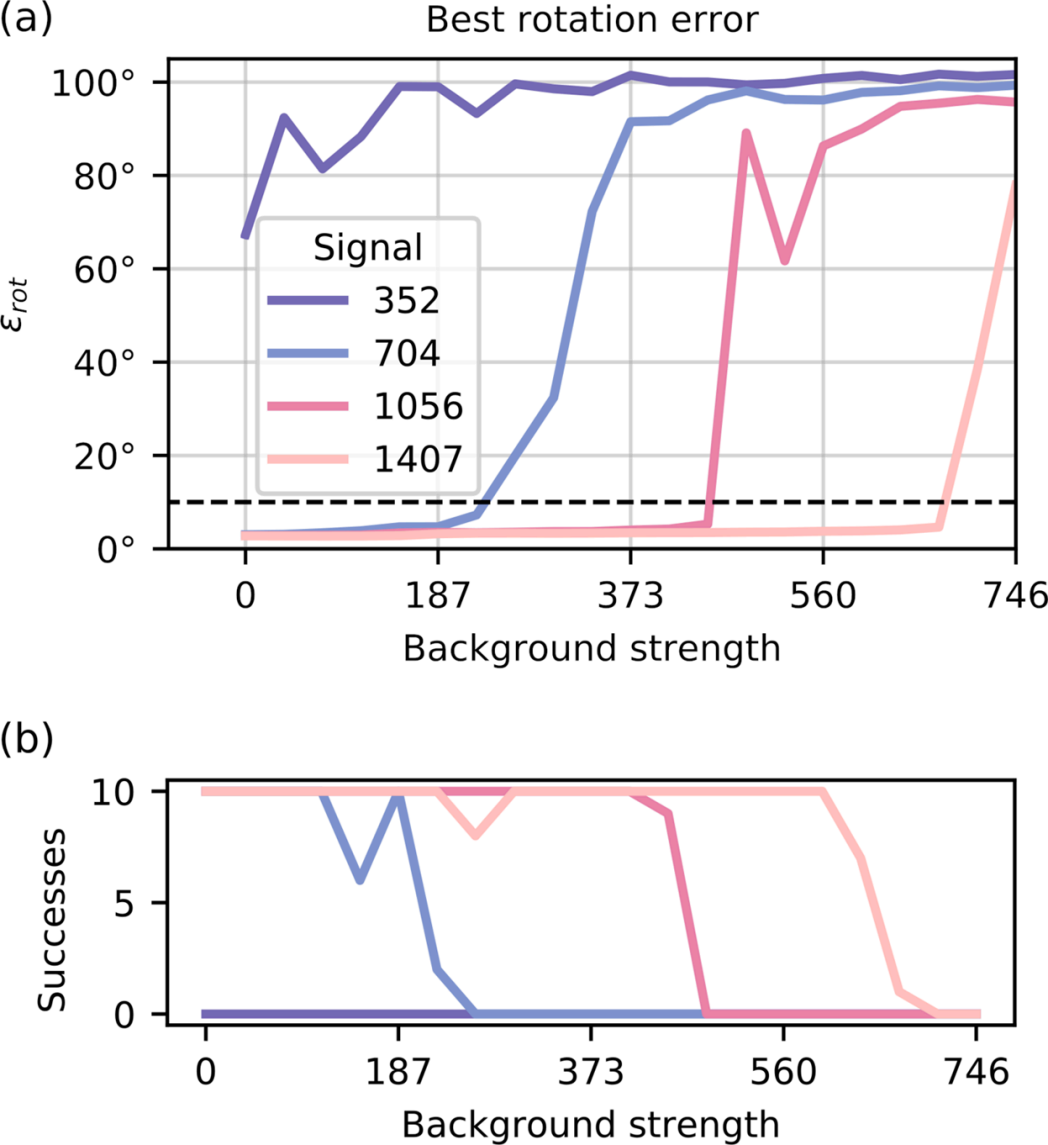
(*a*) The lowest average rotation error ε_rot_ out of ten independent EMC reconstructions, for each combination of signal and background. The dashed line at 10° is the threshold where we consider the intensity reconstruction a success. Both the background strength and the signal strength are measured in average number of photons per diffraction pattern. (*b*) Number of EMC reconstructions that failed to reach 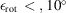. In most cases, reconstructions either failed or succeeded together for a data set, with a small transition region. This shows how remarkably stable EMC is with regard to changes to the random start.

To evaluate each of the 840 independent EMC reconstructions we compared their average rotation errors, ε_rot_, defined in equation (3). The lowest achieved ε_rot_ out of the ten runs is shown in Fig. 1 ▸(*a*), for all of the combinations of background and signal strength. We compare the lowest achieved ε_rot_ to avoid including failed runs, and Fig. 1 ▸ thus represents the best result we achieved with each data set. We see that for the weakest signal ε_rot_ is consistently high, indicating that EMC did not converge to the correct solution regardless of background strength. For the other cases, we see a clear trend, where ε_rot_ is low for weak backgrounds but increases rapidly at a certain background strength.

To investigate the stability of EMC we checked how many of the ten independent intensity reconstructions achieved 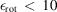
° [see Fig. 1 ▸(*b*)]. The ten independent EMC reconstructions usually either all pass or all fail the threshold. EMC thus seems remarkably stable to changes in the random start, even in the presence of background.

**Figure 2:**
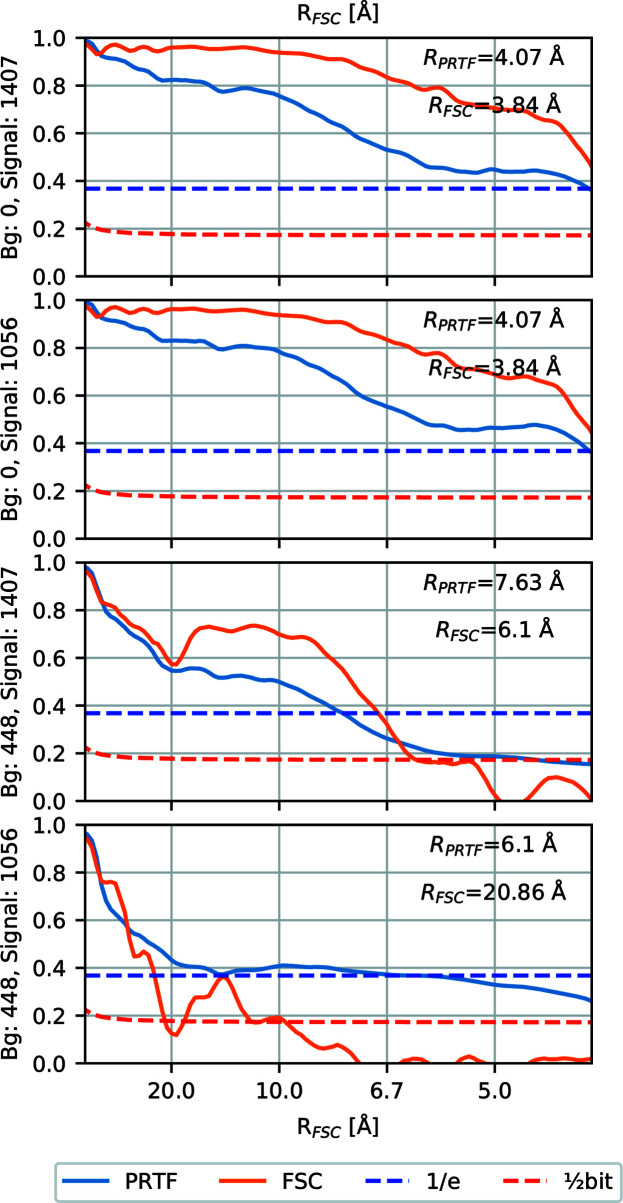
The radial average of the FSC and the PRTF for four different data sets. The signal and background strength are indicated in the average number of signal or background photons per diffraction pattern. The estimated resolution of the reconstruction is shown in the top right of each plot. For the first two plots there is only a minuscule difference between the two signal cases, whereas for the third and fourth plots, with background, the difference between strong and weak signals is clearly apparent. In the final plot, we see a low-resolution reconstruction with an *R*_PRTF_ that is much better than the corresponding *R*_FSC_.

After background subtraction, the phase retrieval algorithm converged to reasonable density maps in most cases. We have two different ways of estimating the resolution of these maps, the PRTF and the FSC. In Fig. 2 ▸ we show the radial average of the PRTF and the FSC, as well as their respective resolutions, for four data sets.

**Figure 3:**
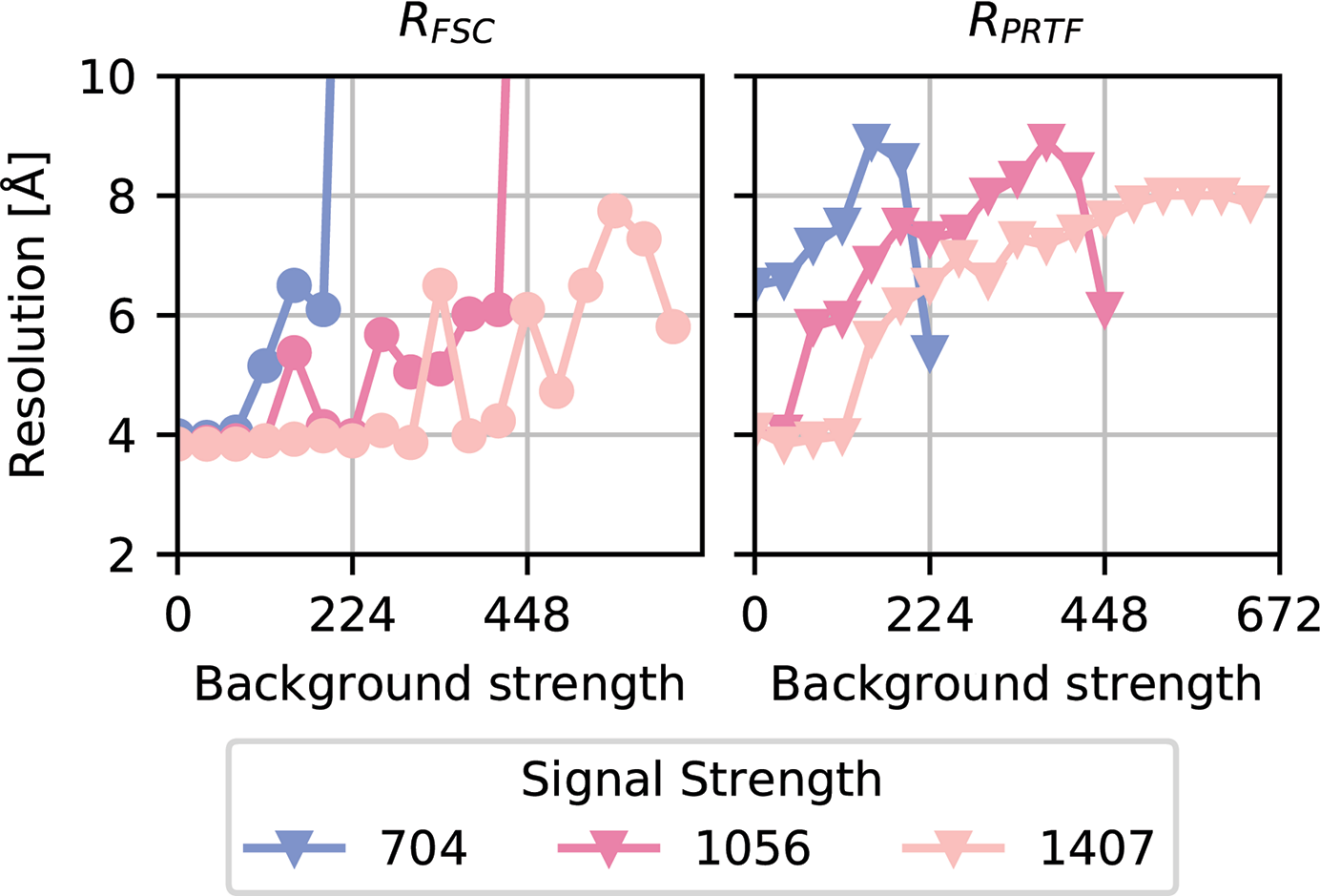
(*a*) The resolution based on the Fourier shell correlation between the average map and the ground truth map, using the ½-bit threshold. (*b*) The resolution based on the PRTF, using the *e*^−1^ threshold. For both measures, the resolution gets worse with increasing background. Note the sudden improvement in *R*_PRTF_ with increasing background for the weaker-signal cases. These correspond to poor reconstructions in (*a*), with high *R*_FSC_, illustrating a situation where the PRTF suggests an exaggerated resolution.

The resolution estimated from the FSC, *R*_FSC_, is shown in Fig. 3 ▸(*a*). The best achieved resolution is 3.84 Å, in the low-to-medium-background regime, which corresponds to the resolution at the edge of the detector This explains why we see a lower bound at this value. In the low-background regime *R*_FSC_ is stable around 4 Å, indicating that background subtraction seems to work well. As the background increases the resolution deteriorates, and for the data sets with weak signal phase retrieval breaks down. Background subtraction also seems less efficient when the signal is weaker.

The resolution estimated from the PRTF, *R*_PRTF_, is shown in Fig. 3 ▸(*b*). It starts at 4 Å and gets progressively worse with increased background up to around 9 Å. After that, phase retrieval fails. Note the unexpected improvement of *R*_PRTF_ of the lower-signal cases with high background. Since we do not see the same improvement in *R*_FSC_ for these cases, we conclude that this is an example of the limitation of the PRTF method where high reproducibility can be achieved without reaching the correct phases.

An interesting comparison can be made between *R*_PRTF_ and *R*_FSC_, because *R*_FSC_ remains at 4 Å for a range of background strengths, while *R*_PRTF_ slowly increases. One interpretation is that there is an offset between *R*_PRTF_ and *R*_FSC_ of around 2–3 Å, which is partially obscured because we are limited to the edge resolution of 3.84 Å in both metrics. If we were to collect data up to a wider scattering angle it could be possible to verify that this trend continues below the 4 Å detector-edge limit. An analogous offset was also observed between *R*_PRTF_ and *R*_FSC_ by Ekeberg *et al.* (2015 ▸).

Since *R*_FSC_ is a measure of how accurately the object was recovered, while *R*_PRTF_ is a measure of reproducibility, *R*_FSC_ is a better measure of success. The fact that *R*_FSC_ is flat is therefore a strong indication that the background subtraction works well. Except for the two outliers with much lower resolution, both estimates stay below 10 Å.

**Figure 4:**
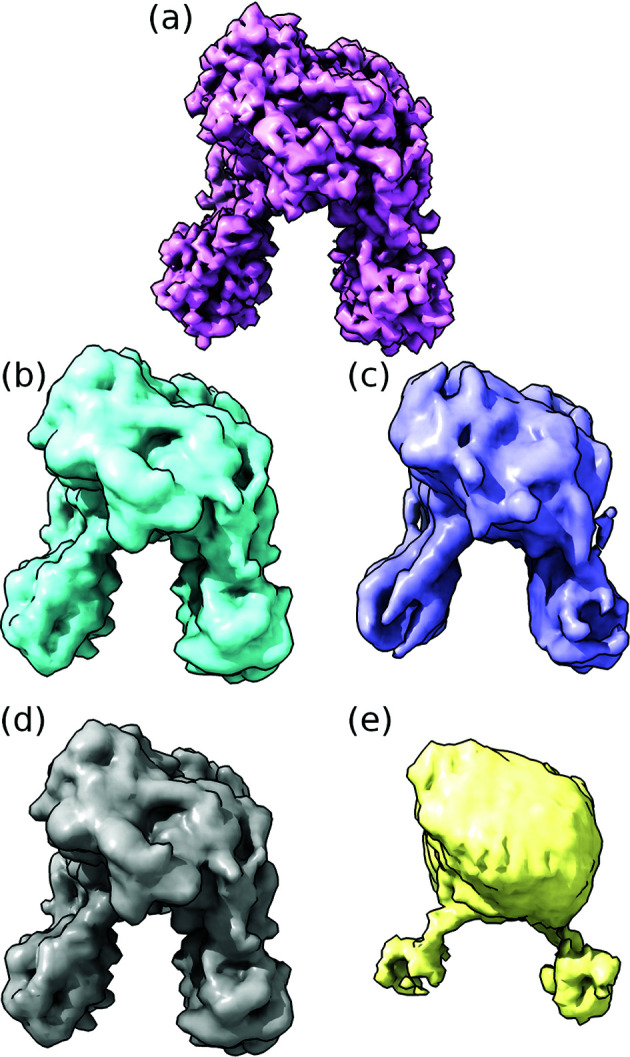
(*a*) The ground truth density of phytochrome, which was used in the calculation of *R*_FSC_, followed by the real-space models of the same reconstructions as in Fig. 2 ▸. (*b*) and (*c*) correspond to the strong-signal case, with an average of 1407 signal photons per pattern, and (*d*) and (*e*) to the weak-signal case, with an average of 1056 signal photons per pattern. (*b*) and (*d*) are from data sets with no background, and (*c*) and (*e*) are from data sets with an average of 448 background photons per pattern. (*b*) and (*d*) look very similar, which matches the similarity in Fig. 2 ▸: both of these had an *R*_FSC_ of 3.84 Å. (*c*) looks significantly worse, with an *R*_FSC_ of 6.1 Å, and (*e*) has a very low resolution of *R*_FSC_ = 20.86 Å.

We visualized the average density maps for a few combinations of background and signal strength in Fig. 4 ▸ to illustrate what the different numerical resolutions correspond to. Note the similarity between Figs. 4 ▸(*b*) and 4 ▸(*d*), and their identical resolutions as seen in Fig. 2 ▸. The low-resolution reconstruction in Fig. 4 ▸(*e*) corresponds to one of the outliers discussed above.

It is difficult to provide a single number for how many signal photons we need to compensate for a given background strength. Depending on the size of the central mask and the distribution of signal and background, the actual number of signal photons can vary by a huge amount. It is more interesting to investigate how the ratio of signal-to-background photons behaves for specific momentum transfers, especially for the high-resolution elements of the reconstruction.

**Figure 5:**
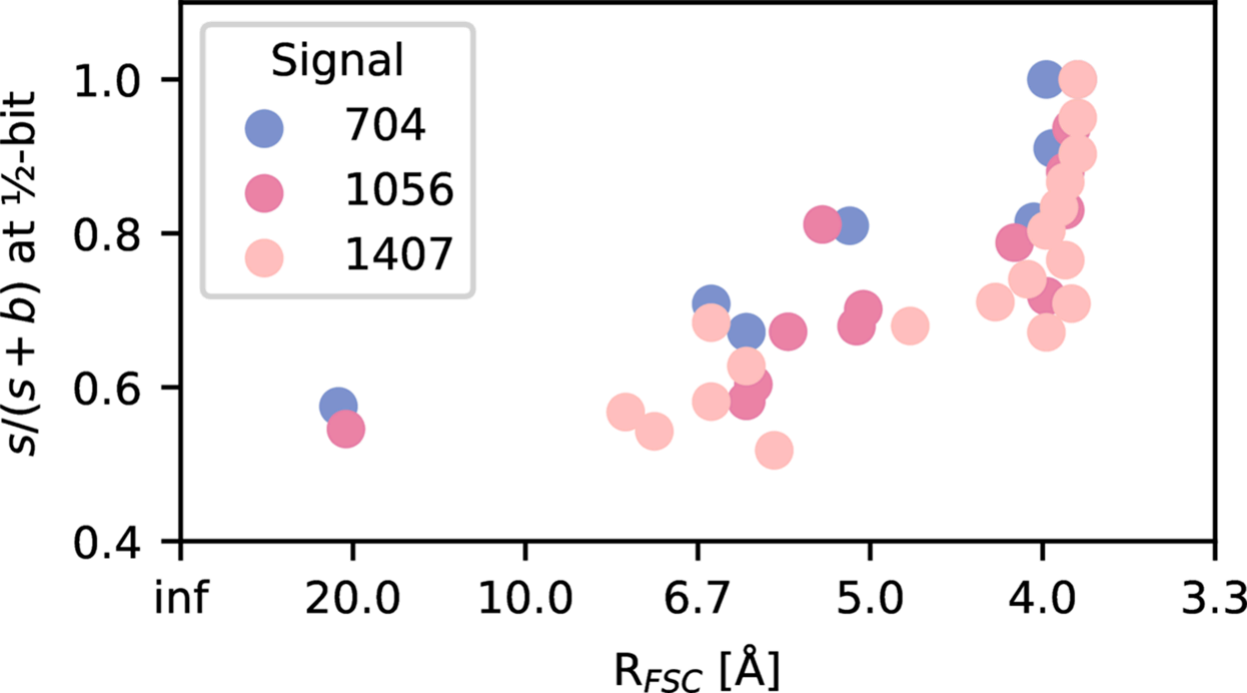
Scatter plot of the signal-to-background ratio *s*/(*s* + *b*) in the resolution shell for which the given reconstruction passes the ½-bit threshold, or the edge of the detector if the FSC never reaches the threshold. Higher-resolution reconstructions require a higher signal-to-background ratio at the maximum recovered resolution. To achieve higher-resolution reconstructions we, therefore, need a stronger signal in general, so that there are data at higher momentum transfers, and the signal needs to be even higher still to compensate for the increase in signal requirement at higher resolutions.

In Fig. 5 ▸ we show the signal-to-background ratio at the momentum transfer for which a given reconstruction’s FSC passed below the ½-bit threshold, which we call the resolution limit according to the FSC. The two dots to the left correspond to the outliers with low-resolution reconstructions. The vertical column of dots on the right corresponds to reconstructions that reached the maximum possible resolution of 3.84 Å. Since the achieved resolution remains constant, but the signal-to-background ratio varies, our interpretation is that *s*/(*s* + *b*) ≈ 0.6 is enough for the resolution limit of the detector’s edge, but more signal does not hurt.

In between these edge cases, we see a weak linear dependence, where higher-resolution reconstructions require a higher signal-to-background ratio at the resolution limit. In other words, to achieve a higher resolution, we not only needed a better signal-to-background ratio, in general, to make the signal clear at higher momentum transfers but also needed it higher still to compensate for this increased signal requirement. We interpret the amount of signal to background we require in a region of the detector as the limiting factor for the resolution of our reconstructions.

## 4. Discussion

We found that for reconstructions to be successful up to a certain resolution the signal-to-background ratio needs to be above 0.6 at the momentum transfer of the target resolution. Somewhat surprisingly, this ratio seems to increase at higher momentum transfers, as can be seen in Fig. 5 ▸. Therefore, in addition to needing more data to compensate for the weaker signal at higher scattering angles, we need a further increase to compensate for these stricter demands that the background places on the analysis. A possible explanation for the need for a higher signal at higher resolutions could be that the signal is partitioned into more reciprocal-space voxels in higher-resolution shells.

A weakness of this investigation is that we consider a specific experimental case in terms of the sample, number of patterns and detector setup. We believe that if the total amount of signal remains constant—distributed over more or fewer patterns—we should see similar results, as long as the number of patterns and photons per pattern are in the hundreds or above. Verifying this assumption could be an interesting future study. If we were to consider a different-sized protein, the study would usually be combined with a modified experimental setup where the choice of photon energy, detector distance and pixel downsampling combine to give speckles of comparable size to what is used in this study. We therefore think that our results are robust to these types of changes.

We also note that the strongest background strength considered is ten times weaker than the experimental measurements, and the signal strength is ten times stronger than what it would be with the fluence currently measured at the European XFEL. The ranges were chosen to expose interesting effects in a setup that would permit structural studies. Improved injector systems where the carrier gas is partially replaced by helium are predicted to provide the needed tenfold decrease in gas background. A background reduction of 80% has already been observed (Yenupuri *et al.*, 2024 ▸). Furthermore, one could reach a ten-times-higher signal strength by studying a heavier particle such as the ribosome.

Another observation is that almost all combinations of signal and background for which EMC converged could also be phased after appropriate background subtraction, although sometimes to a poor resolution. This is somewhat contradictory to our earlier experience that phase retrieval was more challenging than EMC (Lundholm *et al.*, 2018 ▸). A possible explanation would be that the experimental background used here is more stable than the one measured by Lundholm *et al.* or that we have a better knowledge of the average background, which allows a more precise background subtraction.

For phase retrieval, we needed a higher sampling density of the patterns than what was required for a successful orientation recovery. This result was somewhat surprising since both sampling densities were above the theoretical limit of containing twice as many voxels as the size of the sample. It is possible that the phase retrieval failed at sampling densities that are too close to the theoretical limit, owing to imperfections caused by the background noise and inaccurate orientations. It cannot be ruled out that using the same 256^2^ pixel array for orientation recovery would have produced even better results. However, since many reconstructions reached resolutions corresponding to the detector edge, we do not think that any precision lost in this step affects our conclusions.

If readers are judging the feasibility of an experiment, they should consider the expected signal-to-background ratio at the target resolution and estimate if this could be above 0.6. We barely saw any failed EMC runs that passed this condition, and we show in Fig. 5 ▸ that phase retrieval also works here.

## 5. Conclusions

In this paper, we tested how the combination of background and signal strength affects the single-particle imaging analysis pipeline. We found that EMC and phase retrieval were surprisingly robust to the background and that the achieved resolution was only slightly diminished as background strength increased. We also saw that the signal-to-background ratio *s*/(*s* + *b*) was a reasonable measure to predict success and should generally be above 0.6 in any given resolution shell to permit phase retrieval to that resolution. Furthermore, this limit increased slightly in higher-resolution shells. To estimate the signal-to-background ratio in an experiment one can compare data where sample was being injected and data with only buffer, or compare hits with non-hits.

This work highlights the need for improved background environments at XFELs, to take single-particle imaging to the molecular realm. However, background handling should also be a priority for algorithm development, where a deeper understanding of the background might enhance the algorithms, further improving reconstructions.
